# Sensitive Detection of Thiourea Hazardous Toxin with Sandwich-Type Nafion/CuO/ZnO Nanospikes/Glassy Carbon Composite Electrodes

**DOI:** 10.3390/polym13223998

**Published:** 2021-11-19

**Authors:** Mohammed M. Rahman, Md M. Alam, Sulaiman Y. M. Alfaifi, Abdullah M. Asiri, Meser M. Ali

**Affiliations:** 1 Department of Chemistry, Faculty of Science, King Abdulaziz University, P.O. Box 80203, Jeddah 21589, Saudi Arabia; syalfaifikau@gmail.com (S.Y.M.A.); amasirikau@gmail.com (A.M.A.); 2 Center of Excellence for Advanced Materials Research (CEAMR), King Abdulaziz University, P.O. Box 80203, Jeddah 21589, Saudi Arabia; mmalam1@kau.edu.sa; 3 Cellular and Molecular Imaging Laboratory, Department of Neurosurgery, Henry Ford Hospital, Detroit, MI 48202, USA; mali8@hfhs.org

**Keywords:** Nafion/CuO/ZnO NSs/GCE, thiourea sensor probe, differential pulse voltammetry, hydrothermal method, sensitivity, glassy carbon electrode, validation

## Abstract

In this research study, we developed a voltammetric electrochemical sensor probe with a copolymer Nafion (*Sulfonated Tetrafluoroethylene-based Fluoro-polymer*) decorated with hydrothermally prepared sandwich-type CuO/ZnO nanospikes (NSs) onto a glassy carbon electrode (GCE) for reliable thiourea (TU) detection. The detailed characterizations in terms of structural morphology, binding energy, elemental compositions, grain size and crystallinity for synthesized NSs were performed by field emission scanning electron microscopy (FESEM), X-ray photoelectron spectroscopy (XPS), energy-dispersive X-ray spectroscopy (EDS) and X-ray diffraction (XRD) analysis, respectively. The differential pulse voltammetric (DPV) analysis for TU showed good linearity at current-versus-TU concentration on the calibration plot in the 0.15~1.20 mM range, which is defined as a dynamic detection range (LDR) of TU in a phosphate buffer solution. Considering the slope of LDR over the GCE-coated NSs surface area (0.0316 cm^2^), the TU sensor sensitivity (0.4122 µA µM^−1^ cm^−2^) was obtained. Besides this, the low limit (LOD) for TU detection was calculated and found to be 23.03 ± 1.15 µM. The fabricated Nafion/CuO/ZnO NSs/GCE sensor probe was created as a reliable sensor based on reproducibility, interference effect, stability and response time. Real bio-samples were investigated and the results confirm the anticipated reliability of the TU sensor probe. Thus, this is a noble way to develop enzyme-free electrochemical sensors that could be an alternative approach for the detection of chemicals in the field of enzyme-free biosensor development technology.

## 1. Introduction

Within the technological revolution, thiourea (TU) is a compound containing carbon, hydrogen, sulfur and nitrogen widely applied for human and veterinary medicines formulation, agrochemicals production, rubber vulcanization, metal electro-deposition, cleaning agents and food additives [1,2,3]. Besides this, TU performs as a potential agent protecting from browning in horticulture [4]. To inhabit the fruits riping early, as well as breaking seeds and tubers dormancy, TU is used as potential agrochemicals [5]. In addition, it is a preservative of citrus fruits, protecting them from fungi in cold storage conditions [6]. On the other hand, TU is toxic and enlisted as a human carcinogen by the USDHHS (United State Department of Health and Human Services) [7]. Consequently, it is necessary to avoid TU contamination in the environment caused by industrial activities. Further, in humans, the exposure to TU is responsible for the inhibition of the activities of the thyroid, resulting in hypothyroidism syndrome. The positive side is that TU is a potential drug for hyperthyroidism syndrome [8]. Other TU’s effects on humans result in the compound being a reliable measuring tool to check human serum, food, soft drinks, portable and periodical environmental water resources. On the aspect of clinical diagnosis and analysis of the environment, HPLC [9], mercury (II) chloride titration [10], and FTIR [11] are the most frequently applied techniques.

For this purpose, the above-mentioned analytical methods are not suitable for extensive use due to their noticeable disadvantages, such as costly and heavy equipment (HPLC and FTIR), expensive chemical reagents used (HPLC), long analyzing time (HPLC and titrations) and poor sensitivity (FTIR and titrations). To overcome the drawbacks of existing techniques, researchers are trying to invent alternative methods with substantial advantages that are handier and more comfortable to apply. It has been reported that various luminescent probes have been developed for this purpose, such as Au-NPs (gold nanoparticles) and N-GQDs (nitrogen-doped graphene quantum dots), to detect TU via the fluorescent method [12,13]. Besides this, a calorimetric TU sensor based on silver nanoparticles (Ag-NPs) has been constructed for the reliable detection of TU [14]. Moreover, the iridium (III) complex has been used to detect TU in the living cell [15]. Using Ag/Cu nanoparticles, TU has been quantified analytically on an electronic refining bath [16]. Besides, the electrochemical I-V approach has been applied in the development of TU sensors based on Co_3_O_4_/MnO_2_ nanoparticles [17] and SnO_2_/V_2_O_5_ nanomaterials (NMs) [18] on GCEs. Furthermore, the voltammetric electrochemical approach also has been reported for the reliable detection of TU based on Ag-NPs on a graphite electrode and MnO_2_/CNT on a GCE individually [19,20].

Among the electrochemical detection methods of trace biochemical, cyclic voltammetry (CV), impedance spectroscopy (EIS) and differential pulse voltammetry (DPV) are potential tools for researchers to develop sensor probes based on semiconductor metal oxides as sensing elements [21,22]. The attractive surface structure of nanomaterials has improved the sensing performance in terms of sensor sensitivity, dynamic detection range, identical reproducibility in an analysis of analytes and lowest limit of detection with long-term working ability. Remarkably, the p-type and n-type semiconductors and their mixture are a concern for researchers in developing new sensors [23]. The formation of a diode-type junction between p-type and n-type semiconductors in their mixture is the main reason to use them for the improvement of electrochemical sensors [24]. Thus, the CuO-doped ZnO hybrid has shown enhanced conductivity in the sensing of toxic chemicals and biochemicals due to the formation of p(CuO)–n(ZnO) junctions. Recently, the CuO-doped ZnO hybrid has been reported as a potential electrochemical sensor to detect ascorbic acid [25], melamine [26] and 1,2-dichlorobenzene [27], applying the I-V approach. Besides this, CuO-doped ZnO nanorods have been investigated for the development of a non-enzymatic glucose sensor applying a voltammetric electrochemical approach [28,29].

Thus, this study was performed to develop a selective thiourea (TU) sensor based on CuO/ZnO NSs as a sensing substrate deposited on a GCE, where a Nafion copolymer was used as binding glue between CuO/ZnO NSs and a GCE. The assembled sensor probe was investigated to calculate the sensitivity, wide detection range, short response time, good reproducibility and long-term stability. The obtained parameters were deemed to be good and promising results. To establish the reliability, real bio-samples were thoroughly analyzed and the results were found to be acceptable and satisfactory. Thus, this approach could be a new route for the improvement of electrochemical sensors, which might be reliable in terms of the real-time monitoring.

## 2. Experimental Sections

### 2.1. Materials and Tools

Sigma-Aldrich (St. Louis, MO, USA) supplied the analytical grade chemicals, such as Zn(NO_3_)_2_ · 6H_2_O, CuCl_2_ · 2H_2_O, ammonium hydroxide, thieurea (TU) and mono- and di-sodium phosphate, used to perform this study. Additionally, 5% Nafion suspension was obtained from Merck, Germany. FESEM and EDS (JEOL, JSM-7600F, Tokyo, Japan) were performed to evaluate the morphology and elemental compositions of existing atoms in the synthesized CuO/ZnO NSs. The elemental ionization and binding energy were investigated by the implementation of the XPS analysis based on Thermo-Scientific-XPS with a radiation of A1-k-ɑ1 having a beam size of 300 µm worked at 200 eV. The grain size and crystallinity of the unit cell in the prepared nanomaterials were investigated through the analysis of X-ray diffraction studies. The Metrohm-Potentiostat/Galvanostat (Auto-lab), a high-performance modular instrument, was used in this study for assembling the TU sensor probe.

### 2.2. Preparation of CuO/ZnO NSs

The analytical grade CuCl_2_ · 2H_2_O and Zn(NO_3_)_2_ · 6H_2_O acquired from Sigma-Aldrich (St. Louis, MO, USA) were the major constituting chemicals in the preparation of CuO/ZnO nanomaterials and were applied using the hydrothermal method. The hydrothermal method is a very good technique to prepare crystalline nanomaterials. It is used for the synthesis of binary metal oxides of distinct shapes and sizes. In this approach, 0.1 M CuCl_2_ · 2H_2_O and Zn(NO_3_)_2_ · 6H_2_O deionized water solutions were put in two separate 100 mL sized volumetric flasks. Then, 50 mL of the prepared solution from each 100 mL flask was poured in a 200 mL beaker, which was subsequently placed in a Teflon autoclave. After that, 0.1 M NH_4_OH was added into the cell in a dropwise manner for raising the solution’s pH to around 10.5. It should be noted that the Zn^2+^ and Cu^2+^ ions started to precipitate in the alkaline environment and formed a nucleus of crystal formation in the autoclave after heating was set at 90 °C. On further precipitation of Cu(OH)_2_ and Zn(OH)_2_, a big crystal of Cu(OH)_2_ and Zn(OH)_2_ formed. It was assumed that, at this high alkaline environment (pH = 10.5), all metal ions (Zn^2+^ and Cu^2+^) were co-precipitated as previous authors reported [30]. The cell was kept at these conditions for 5 h necessary for the completion of the precipitation. The supposed participation reactions involved in this synthesized process are assumed below (1)–(4):

NH_4_OH _(s)_ → NH^4+^_(aq)_ + OH^−^ _(aq)_ (1)

Zn(NO_3_)_2 (s)_ → Zn^2+^_(aq)_ + 2NO^3−^ _(aq)_ (2)

CuCl_2 (s)_ → Cu^2+^_(aq)_ + 2Cl^−^ _(aq)_ (3)

Zn^2+^_(aq)_ + Cu^2+^_(aq)_ + 4OH^−^_(aq)_ + nH_2_O → Zn(OH)_2_ · Cu(OH)_2_ · nH_2_O ↓ (4)

After that, the precipitated mass of nanocrystals was separated by filtration and successively washed using (deionized) water. Subsequently, it was dried at 120 °C by keeping it in an oven overnight. Later, it was calcined by applying a high temperature of around 500 °C for the five successive hours by having pure oxygen flow into a muffle furnace. It was considered that all the dry metal oxides were oxidized to ZnO and CuO as shown in the following reaction scheme (5):

Zn(OH)_2_ + Cu(OH)_2_ → ZnO/CuO + 2H_2_O (5)

### 2.3. GCE Modification by CuO/ZnO NSs

A commercial GCE (0.0316 cm^2^) was modified by the deposition of a thin layer of CuO/ZnO NSs and the resultant modified GCE was utilized as the sensing electrode known as the working electrode of the TU sensor. For this purpose, the synthesized CuO/ZnO NSs (5.0 µg) were made slurry in ethanol (1.0 mL) and subsequently used to form an NS layer to coat the GCE’s flat part. Next, the sensor was dried by keeping it in the laboratory for several minutes. The sensor stability in the sensing performance is a predetermined requirement for this electrochemical study. Therefore, 5% Nafion (1.0 µL) was applied on the dry NS-modified GCE and used as binding glue of the NS layer on the GCE. Then, it was again dried by keeping it at 35 °C for an hour in an oven. Finally, a Ag/AgCl column electrode (containing the saturated KCl solution), Nafion/CuO/ZnO NSs/GCE and a Pt wire were used to connect with Auto-lab performing as reference, working and counter electrodes, respectively. The target biochemical thiourea was thinned using deionized water to form several solutions at a range of 0.15~1.20 mM and was used for the voltammetric electrochemical analysis. The monosodium and disodium phosphate were used for adjusting the buffer’s pH values in the 5.7~8.0 range. The volume of buffer solution taken to execute the analysis was 30 mL and was kept constant throughout the study. The LOD was calculated with the following Equation (6):

LOD = 3 × SD/(Slope of the calibration plot)(6)

where SD is the standard deviation of the blank response.

## 3. Results and Discussion

### 3.1. Characterization of Synthesized CuO/ZnO NSs by FESEM and EDS

**Figure 1:**
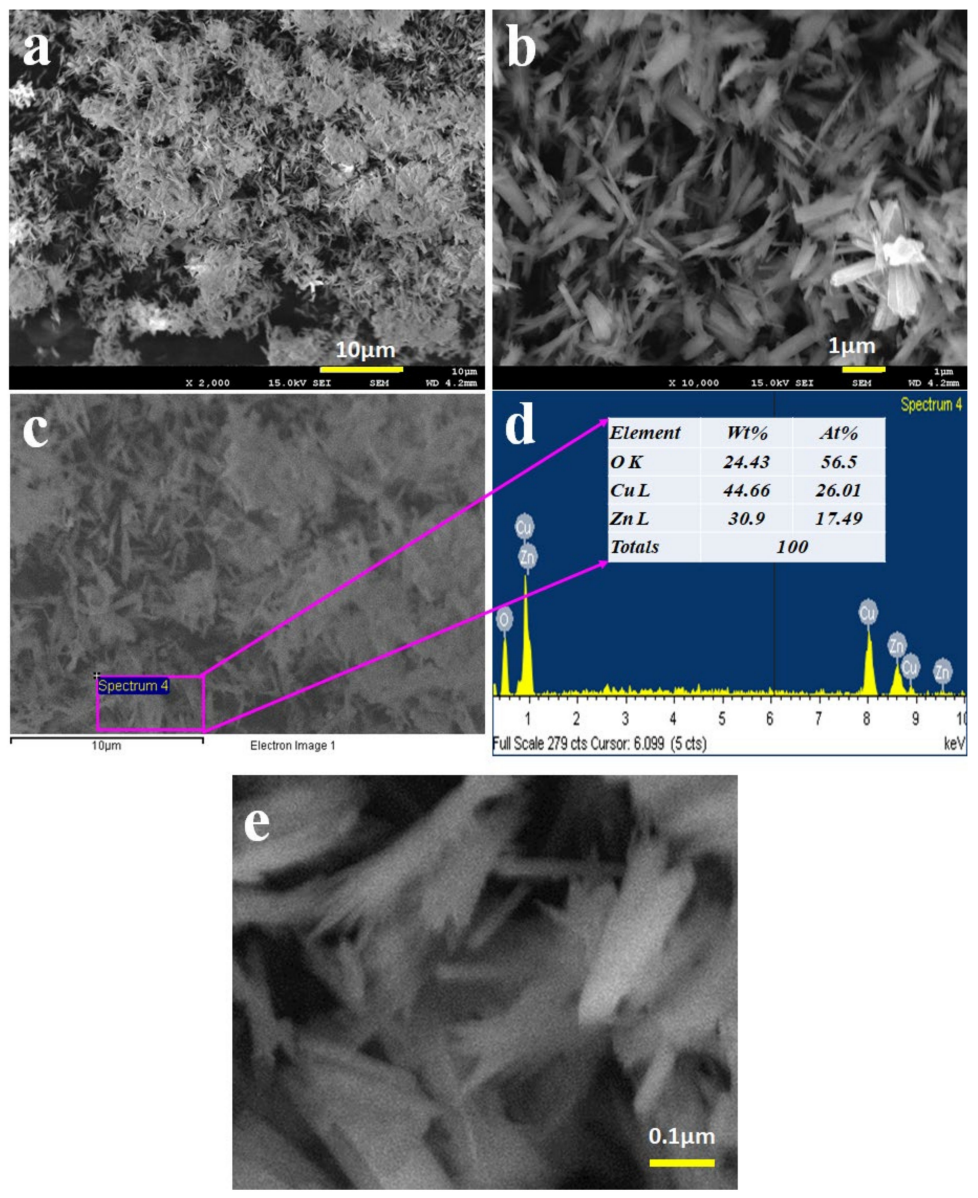
Structural and compositional analyses of CuO/ZnO nanomaterials. (**a**,**b**) Exploration of the low and high magnified images of the prepared nanomaterials captured by FESEM. (**c**) EDS-captured image of CuO/ZnO NSs. (**d**) Compositional analysis of CuO/ZnO NSs in a selected area was performed by EDS investigation. (**e**) Magnified image of CuO/ZnO NSs.

**Figure 2:**
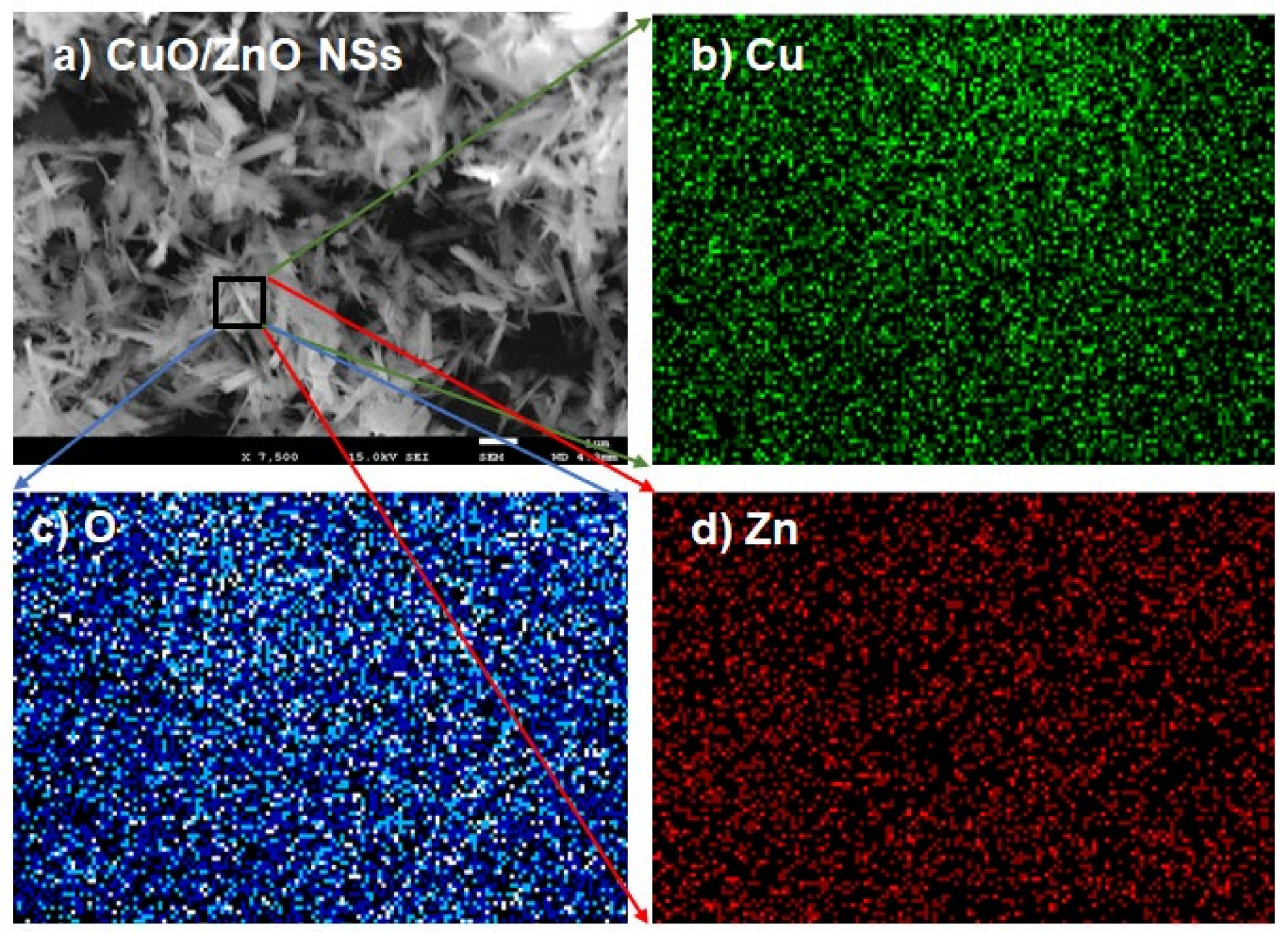
Elemental mapping of CuO/ZnO nanospike materials. (**a**) SEM image of NSs. (**b**) Elemental mapping of the copper element. (**c**) Elemental mapping of the oxygen element. (**d**) Elemental mapping of the zinc element.

The CuO/ZnO NSs were prepared in a pH 10.5 medium by applying the hydrothermal method. The morphology of the CuO/ZnO nanomaterials was accomplished by the execution of FESEM and EDS analyses, as shown in Figure 1. From the illustration, the ZnO and CuO metal oxides aggregated regularly to each other to form spike structures with distinct lengths and diameters as explored in Figure 1a,b. A similar reflection was obtained from the magnified image obtained from the EDS analysis as in Figure 1c. From both FESEM and EDS investigations, it is clearly shown that the prepared nanomaterials can be considered as nano-spikes (NSs). The atomic compositions evaluated by EDS, as in Figure 1d, are 24.43% O, 44.66% Cu and 30.90% Zn as weight. It was also evidenced in co-doped CuO/ZnO NSs. Therefore, it was confirmed that the synthesized NSs contained only the Cu, Zn and O elements—no impurities were identified. A magnified image of FESEM for CuO/ZnO NSs is presented in Figure 1e, where a spike-like structure is found in the image. It is confirmed that the nanostructure of CuO/ZnO NSs is composed of spike-like structures throughout the material. The elemental mapping of CuO/ZnO NSs was performed and is presented in Figure 2a–d. The elemental distribution of each element (Cu, O and Zn) was measured separately and is presented in Figure 2b–d, respectively. Here, it can be observed that the elements are properly distributed among the nanospikes.

### 3.2. XRD Analysis of CuO/ZnO NSs

**Figure 3:**
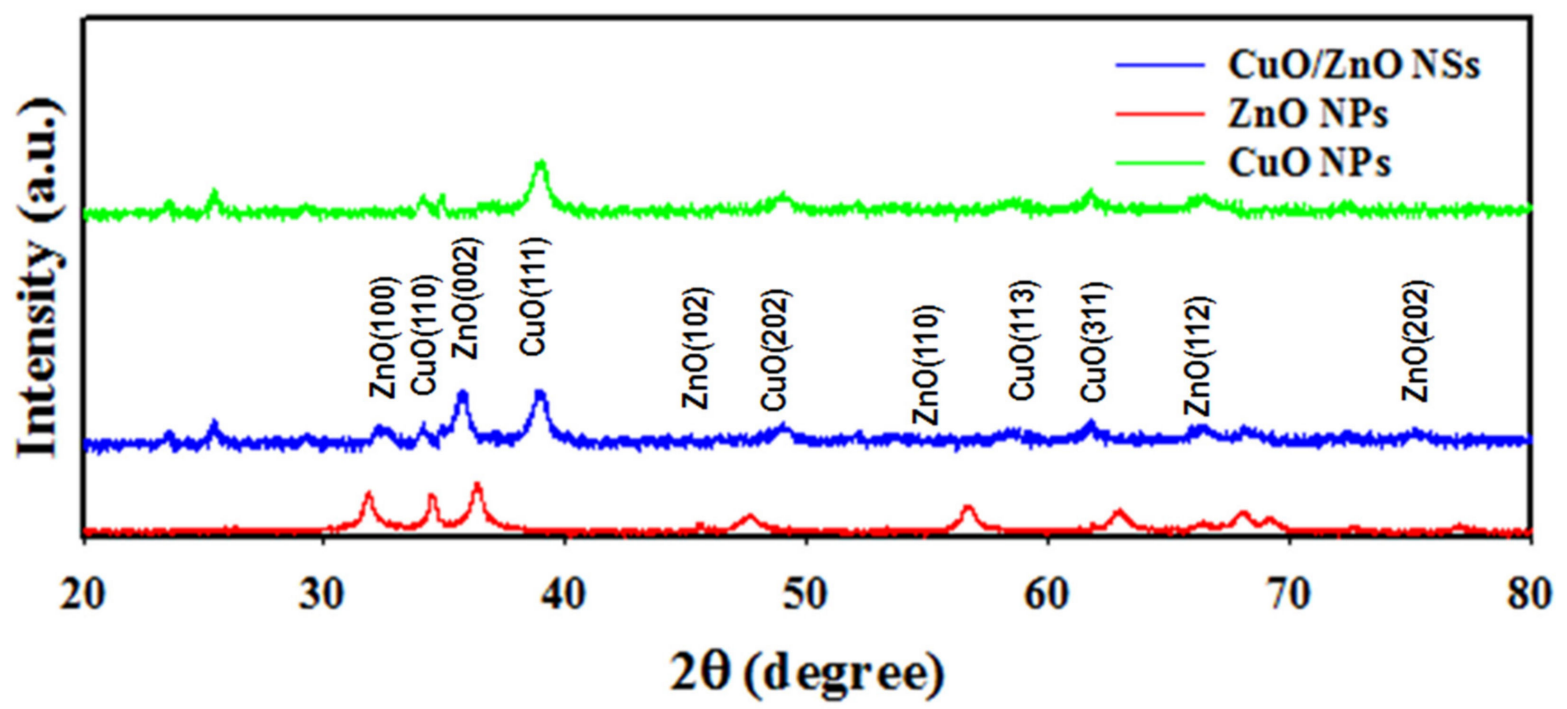
The powder X-ray diffraction analysis of CuO/ZnO NSs.

The X-ray diffraction analysis of the synthesized CuO/ZnO NSs was performed by applying the angle 2θ = 20~80° represented in Figure 3. It was perceived that the NSs consisted of CuO and ZnO only. No impurities were unidentified in this analysis. As pictured in Figure 3, the plans of (110), (111), (202), (113) and (311) of CuO are perceived and identified by JCPDS no. 0045-0937 [31,32]. Besides this, the ZnO crystalline unit cell as (100), (102), (002), (110), (112) and (202) are shown in Figure 3, which matched with JCPDS no. 01-007-2551 [33,34].

Using the Debye–Scherrer formula illustrated in Equation (7), the nanosheet size was measured to be 12.10 nm considering the peak of ZnO (002).

D = 0.9 λ/(βcosθ)(7)

where λ = wavelength (X-ray radiation at 1.5418 °A) and β = the full width of the peak at the half (FWHM) position at the diffracted θ angle. The X-ray diffraction analysis of NSs confirms the existence of CuO and ZnO, which is completely in agreement with the results obtained from the FESEM and EDS analyses.

### 3.3. XPS Analysis of CuO/ZnO NSs

**Figure 4:**
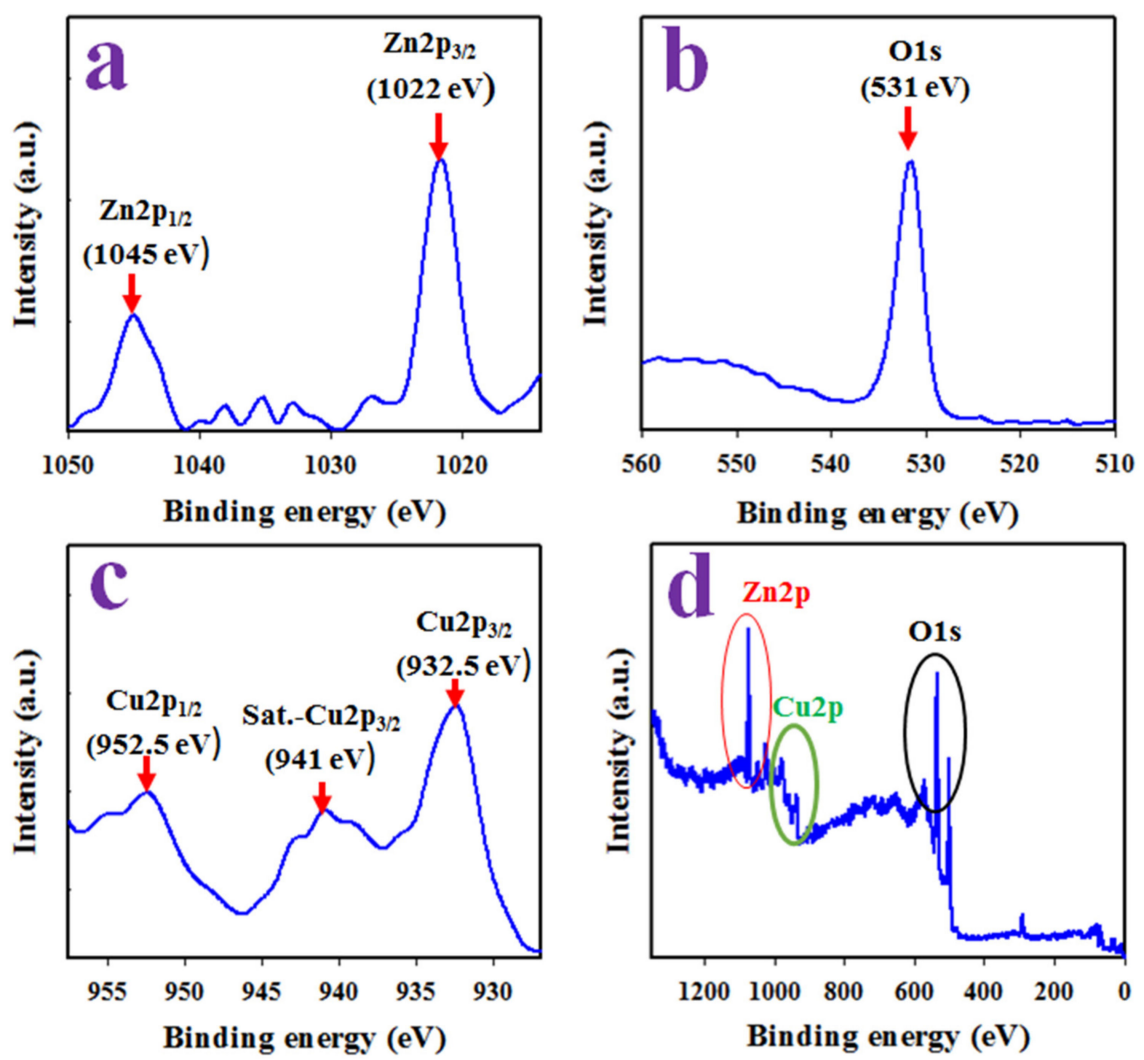
(**a**) The spin orbitals of Zn2p level. (**b**) XPS spectra of O1s level. (**c**) Cu2p spin orbital. (**d**) Full XPS spectrum of prepared CuO/ZnO NSs.

Figure 4 represents the XPS orbitals of the synthesized CuO/ZnO NSs and consists of Zn2p, Cu2p and O1s orbitals only; the peaks correlated with impurities are unidentified. As shown in Figure 4a, the Zn2p orbital is divided into the Zn2p_3/2_ and Zn2p_1/2_ spin orbitals located around 1022 and 1045 eV and having the spin separation of 23 eV, a characteristic value for 2+ oxidation of Zn in the prepared CuO/ZnO NSs and identified by previous articles on ZnO [35,36,37,38]. Figure 4b presents a peak at 531 eV, indicating an O^2−^ ion associated with the Cu–O and Zn–O bonds in the CuO/ZnO NSs [39,40]. Besides this, two spin orbitals of the Cu2p level can be seen in Figure 4c located at 932.5 and 952.5 eV of the Cu2p_3/2_ and Cu2p_1/2_ spin orbitals, correspondingly, and separated by 20 eV. An additional satellite peak located at 941 eV was detected as a Cu2p_3/2_ orbital. Therefore, the main and satellite peaks were both confirmed 2+ oxidation of Cu in the prepared samples [41,42,43]. This XPS study results are completely in agreement with the results obtained from the XRD, FESEM and EDS analyses and provide the evidence of the existence of Cu^2+^, O^2−^ and Zn^2+^. Therefore, it can be concluded that the prepared NSs consisted of co-doped of CuO and ZnO only.

### 3.4. Electrochemical Characterization of Nafion/CuO/ZnO NSs/GCE

**Figure 5:**
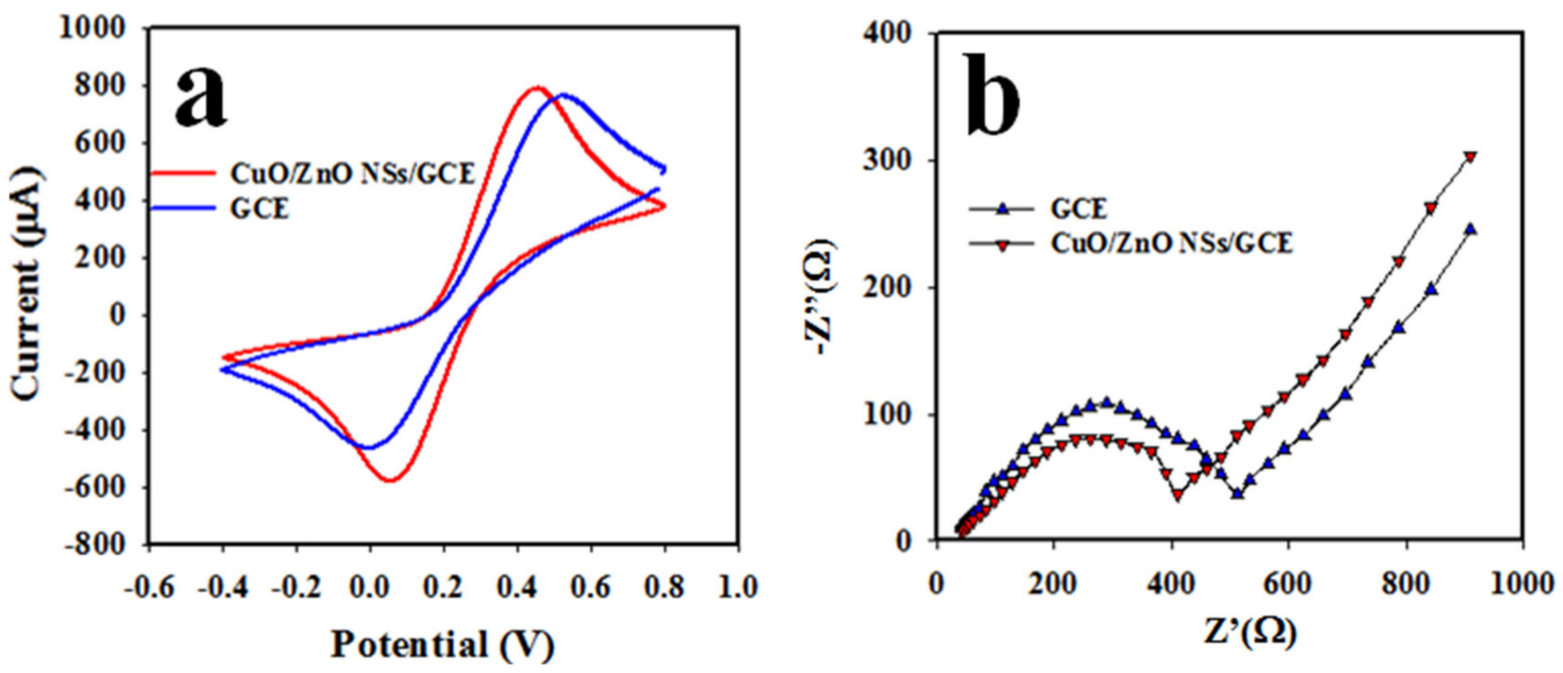
Electrochemical analysis of Nafion/CuO/ZnO NSs/GCE sensor probe. (**a**) The cyclic voltammetry of bare and CuO/ZnO NS-modified GCE in 0.1 mM ferricyanide/ferrocyanide [K_3_Fe(CN)_6_] in a pH 7.0 buffer and (**b**) the electrochemical impedance spectroscopy (EIS) of bare and CuO/ZnO NS-modified Nafion/GCE in 0.1 mM ferricyanide/ferrocyanide [K_3_Fe(CN)_6_] in a pH 7.0 buffer.

The electron movement of the Nafion/CuO/ZnO NSs/GCE working electrode was estimated by performing a cyclic voltammetric (CV) analysis of a 0.1 mM solution of ferricyanide/ferrocyanide [K_3_Fe(CN)_6_] couple in a pH 7.0 buffer, as shown in Figure 5a. The oxidation as well as reduction peak current of the modified GCE were perceived at +0.45 and +0.08, respectively, as illustrated in Figure 5a, with a separation potential of +0.37 V. In contrast, the bare GCE revealed the low oxidation and reduction peak current to be located at +0.50 and 0 V, respectively, with a wider peak separation of +0.50 V, compared with the coated GCE (+0.37 V). Thus, it can be predicted that the high electron mobility for Nafion/CuO/ZnO NSs/GCE would perform optimally in the analysis of an analyte electrochemically for its narrow oxidation–reduction peak separation potential (∆Ep), as presented in Figure 5a. This statement can be confirmed by previously reported articles on electrochemical detection of the analyte [44].

Conductance is significant parameter for the Nafion/CuO/ZnO NSs/GCE working electrode, which is necessary to assess the oxidation/reduction capability of an analyte by the assembled sensor probe. Therefore, the electrochemical impedance spectroscopy (EIS) in 0.1 mM ferricyanide/ferrocyanide [K_3_Fe(CN)_6_] couple in a pH 7.0 buffer solution was performed with the working electrode based on the Nafion/CuO/ZnO NSs/GCE, as illustrated in Figure 5b. From the EIS in Figure 5b, the semi-circle diameter is smaller with the Nafion/CuO/ZnO NSs/GCE than with the bare GCE, which provides the evidence for the smaller charge-transfer resistance (R_ct_) of the assembled working electrode. Therefore, it is logical to assume that the modified Nafion/CuO/ZnO NSs/GCE working electrode has more capacity to enhance the charge-transfer in an electro-catalytic reaction. This concept has also been reported elsewhere [45,46].

**Figure 6:**
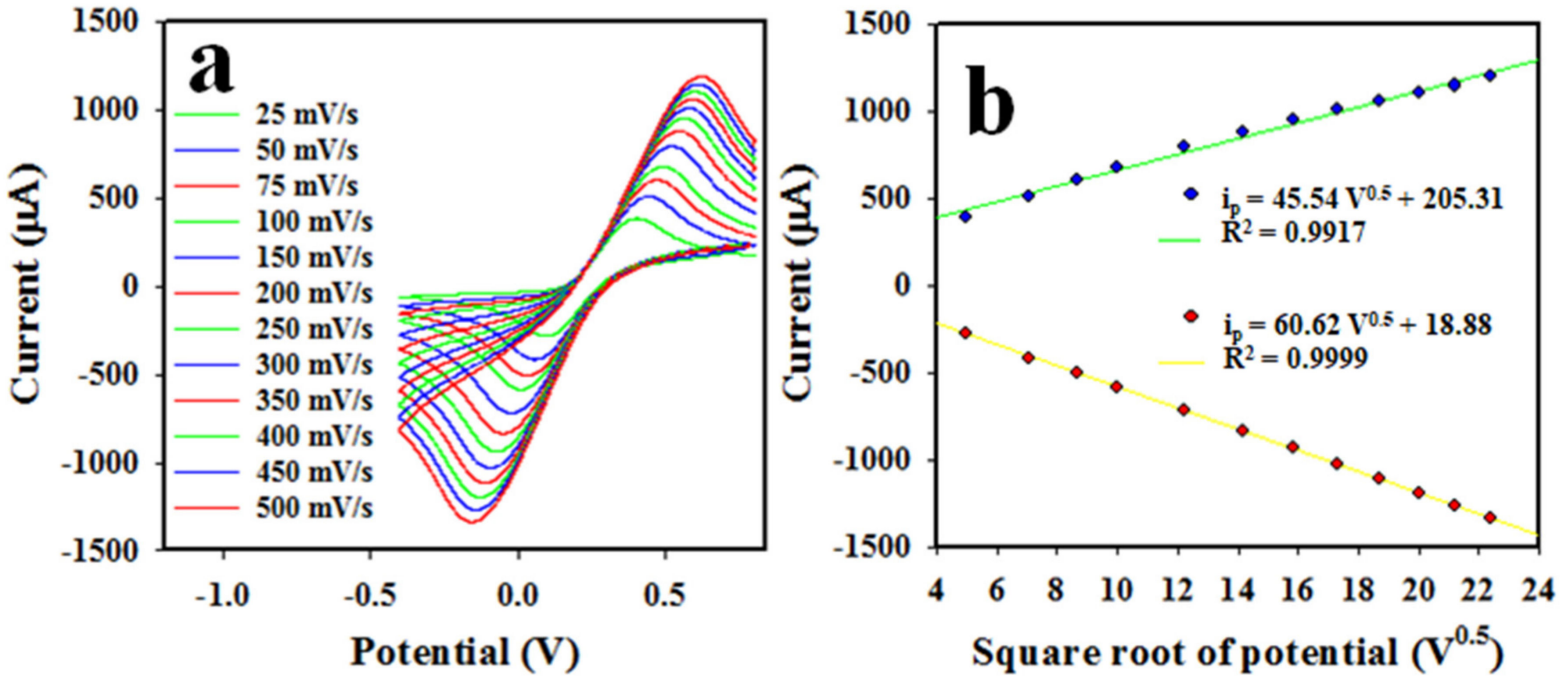
Electrochemical investigation of Nafion/CuO/ZnO NSs/GCE. (**a**) Scan rate of assembled working electrode based on CuO/ZnO NSs in CV analysis of 0.1 mM ferricyanide/ferrocyanide, in a pH 7.0 buffer. (**b**) V^1/2^versus i_p_ (oxidation/reduction).

Figure 6a below illustrates the scan rate (SR) of the Nafion/CuO/ZnO NSs/GCE electrode in the cyclic voltammetric analysis of 0.1 mM ferricyanide/ferrocyanide [K_3_Fe(CN)_6_] in a pH 7.0 buffer solution. As shown in Figure 6a, the peak currents for oxidation and reduction were individually positioned at +0.45 V and +0.08 V and were found to increase and decrease in a linear manner, respectively, as the SR was applied in a range of 25~500 mV/s. Therefore, a calibration curve was executed by plotting the obtained peak current for the oxidation (i_p_) and reduction (i_p_) of 0.1 mM ferricyanide/ferrocyanide [K_3_Fe(CN)_6_] as square roots of SR^0.5^ versus i_p_, as proven in Figure 6b, which can be expressed as i_p_ = 45.54V^1/2^ + 205.31 (regression coefficient, R^2^ = 0.9917) for oxidation and i_p_ = 60.62V^1/2^ + 18.88 (regression coefficient, R^2^ = 0.9999) for the reduction process. As shown in Figure 6b, the current versus V^1/2^ had good linearity for both oxidation and reduction reactions of ferricyanide/ferrocyanide in the buffer phase and it can be concluded that these reactions are followed by a diffusion-controlled process.

### 3.5. Electrochemical Detection of Thiourea with Nafion/CuO/ZnO NSs/GCE Sensor Probe

**Figure 7:**
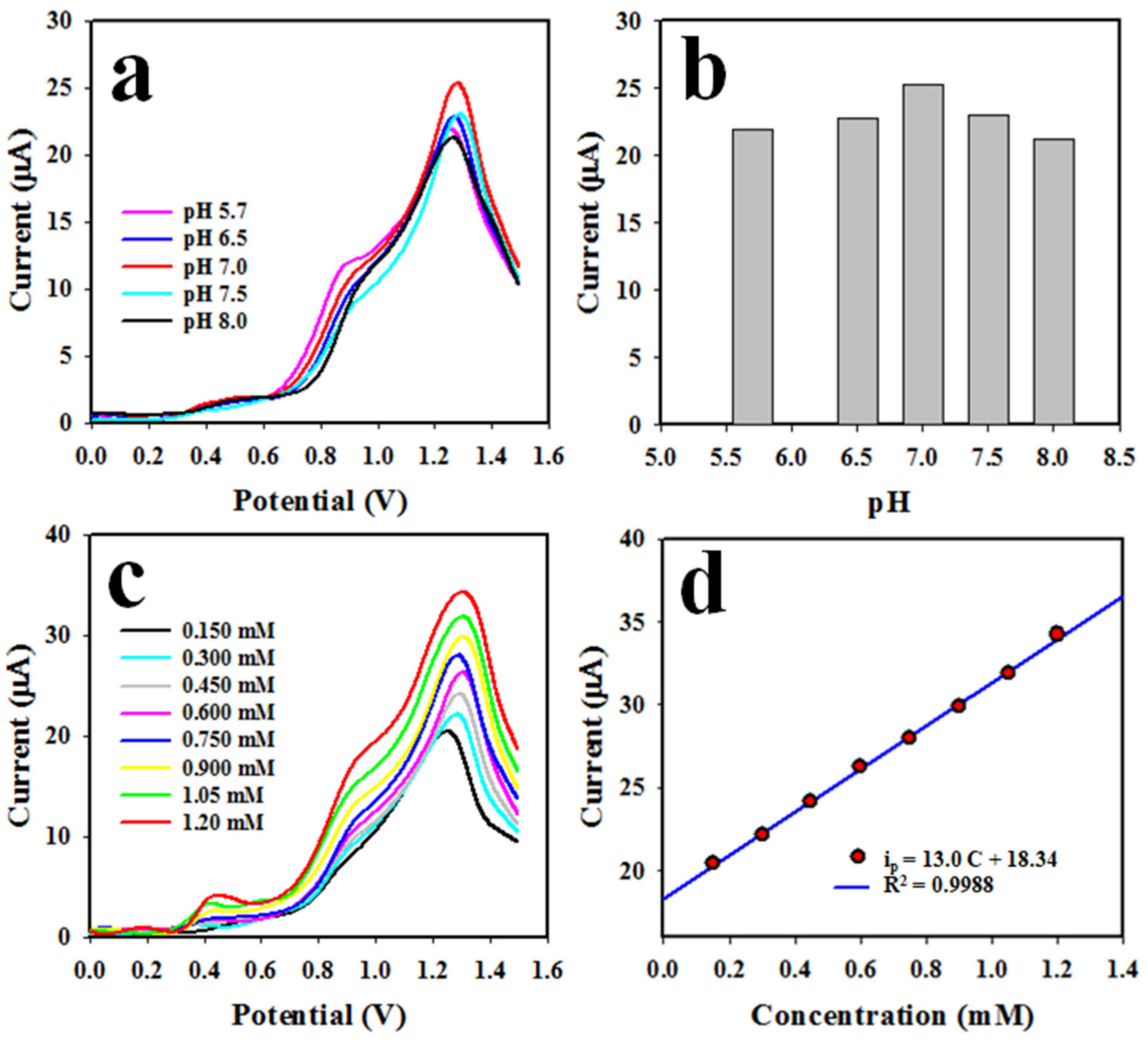
(**a**) The effects of pH on the DPV analysis of TU, (**b**) the bar diagram of the effects of pH in the electrochemical analysis of TU, (**c**) the DPV analysis of TU at pH 7.0 using a low-to-high concentration range and (**d**) TU sensor calibration.

The differential pulse voltammetric (DPV) method was applied to the analysis of TU. The assembled working electrode based on Nafion/CuO/ZnO NSs/GCE was used to detect TU in a 0.60 mM pH 7.0 buffer. Figure 7a presents the optimization of the pH (5.7~8.0) value of the buffer solution necessary to obtain the maximum peak current for the oxidation of TU using a 0.60 mM concentration. It demonstrates that the highest oxidation peak current is obtained with a pH of 7.0 in the analysis of TU when applying the DPV electrochemical method, which is illustrated with the bar diagram in Figure 7b. In the next stage, a range (0.15~1.20 mM) of TU solution in the pH 7.0 buffer was subjected to the electrochemical analysis presented in Figure 7c. It can be observed that the peak current for the oxidation of TU was found to be increased from a low to a high concentration of TU. As it can be observed, the current density is enhanced with the increase in the concentration of the analyte (TU), thus the electrochemical oxidation of the analyte occurs. To calibrate this TU sensor, the peak point currents in the analysis of TU were plotted as current versus TU concentration, shown in Figure 7d. Thus, the resulting straight line is fitted with i_p_(µA) = 13.0 C(mM) + 18.4 and the regression coefficient R^2^ = 0.9988 at a 0.15~1.20 mM TU concentration; 0.15~1.20 mM is defined as the maximum detection range (LDR) of TU, which can be performed by the proposed TU sensor based on the Nafion/CuO/ZnO NSs/GCE. Noticeably, the attained LDR is very wide for the analysis of TU.

The slope of LDR over the surface area of the GCE (0.0316 cm^2^) was used to measure the sensor sensitivity, which was found to be 0.4122 µA µM^−1^ cm^−2^, a value we consider satisfactory. The lower limit (LOD) of TU detection was calculated as 23.03 ± 1.15 µM, which we consider satisfactory. The LOD was calculated with the following Equation (8):

LOD = 3 × SD/(Slope of the calibration plot)(8)

where SD is the standard deviation of the blank response.

**Figure 8:**
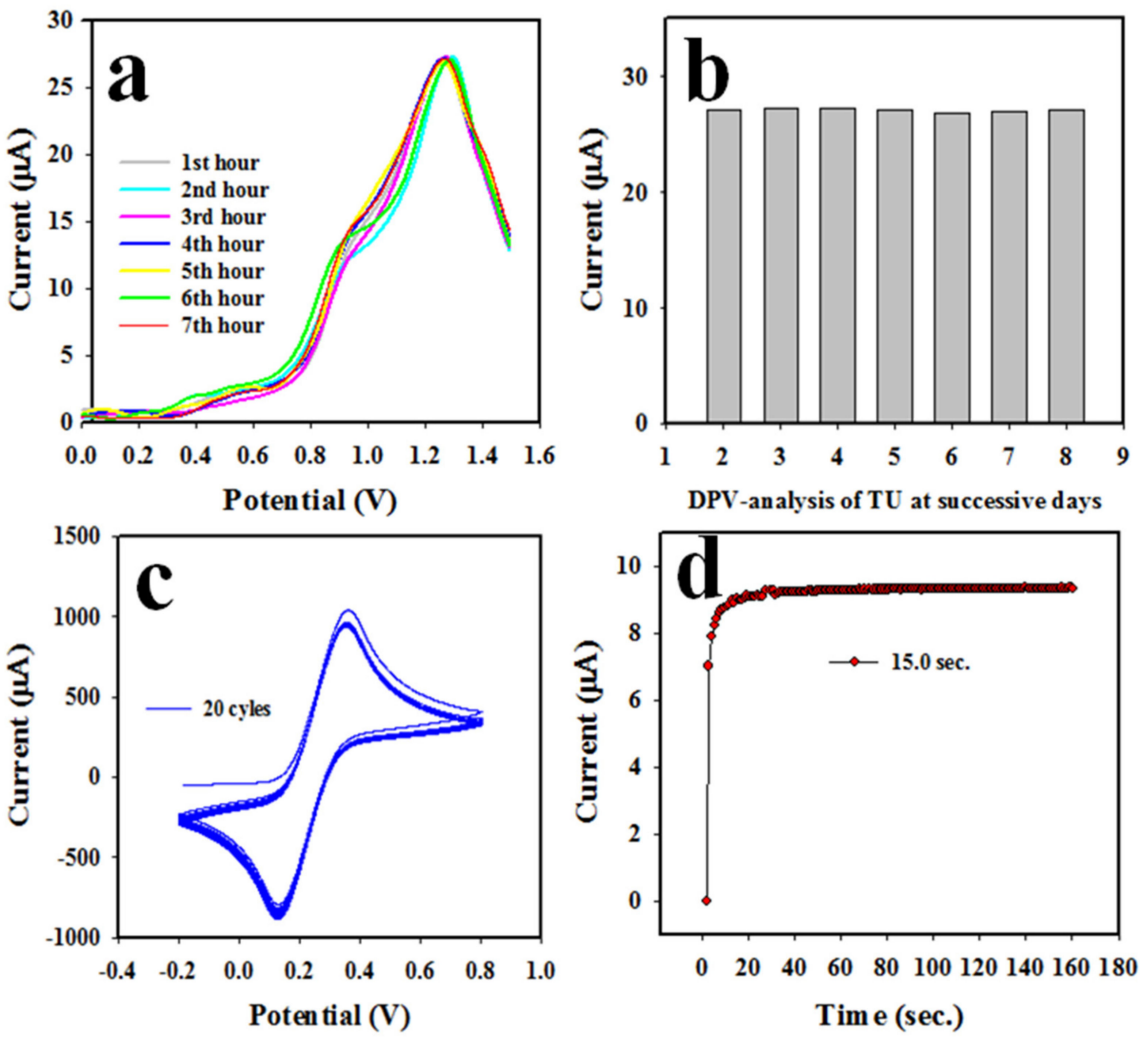
Optimization of sensor probe of Nafion/CuO/ZnO NSs/GCE. (**a**) Reproducibility of TU sensor, (**b**) reproducibility represented in a bar diagram, (**c**) stability of TU sensor and (**d**) response time.

The stability of the NS-deposited layer on the GCE in the TU DPV analysis is a requirement to develop a reliable electrochemical sensor. Therefore, the reproducibility of the TU sensor using the Nafion/CuO/ZnO NSs/GCE was tested using 0.60 mM TU in a pH 7.0 buffer medium; the obtained data are shown in Figure 8a,b (bar diagram). As can be seen in Figure 8a, the obtained outcomes are completely undistinguishable and identical. From Figure 8a,b, we can infer that the electrochemical sensor with the Nafion/CuO/ZnO NSs/GCE working electrode can analyze TU reliably. The stability of this TU sensor is another essential criteria (parameter). The experiment was performed using 0.1 mM ferricyanide/ferrocyanide in a pH 7.0 phosphate buffer, as it can be seen in Figure 8c. Therefore, it can be considered that the assembled TU sensor is quite stable in its performance at 20 cycles of the CV analysis. To measure the sensor’s performing efficiency, a curve of current versus time was plotted (shown in Figure 8d) using 0.60 mM TU in a pH 7.0 buffer medium. It can be clearly seen that the current-versus-time plot becomes steady at around 15 s. Thus, the proposed TU sensor needs 15 s to perform a voltammetric analysis of TU detection, a value which indicates high efficiency.

**Figure 9:**
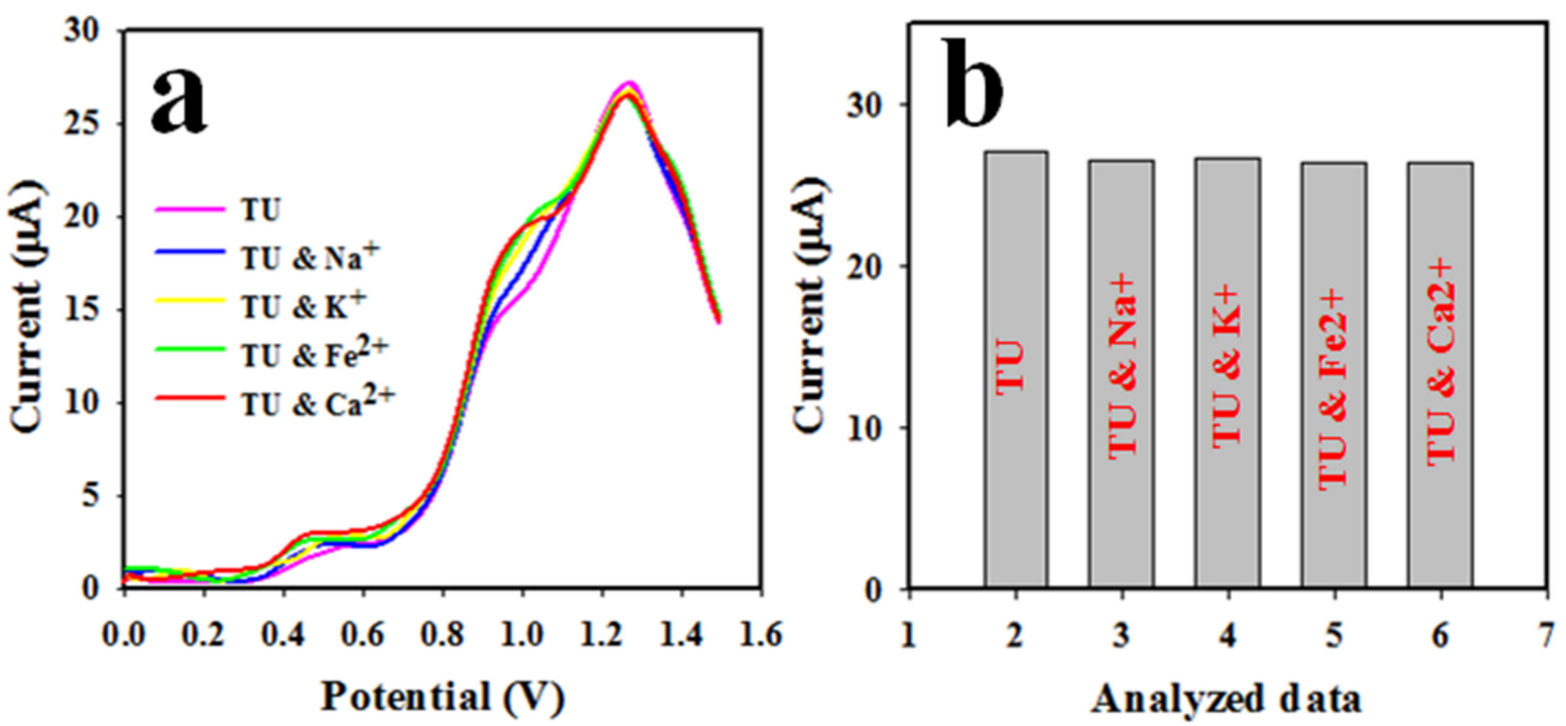
(**a**) The assessment of the interference effect of TU sensor in the presence of common electrolytes and (**b**) bar diagram of the interference effect.

As is known, human blood serum and urine contain various common metal ions, such as Ca^2+^, Na^+^, Fe^2+^, K^+^, etc. Therefore, TU was analyzed by applying the DPV method in the presence of these metal ions in a pH 7.0 buffer solution and 0.6 mM TU concentration; the resultant outcome is shown in Figure 9a,b (bar diagram). As pictured in Figure 9, the common electrolytes did not show any measurable interference in the analysis of TU when applying the DPV method.

**Scheme 1:**
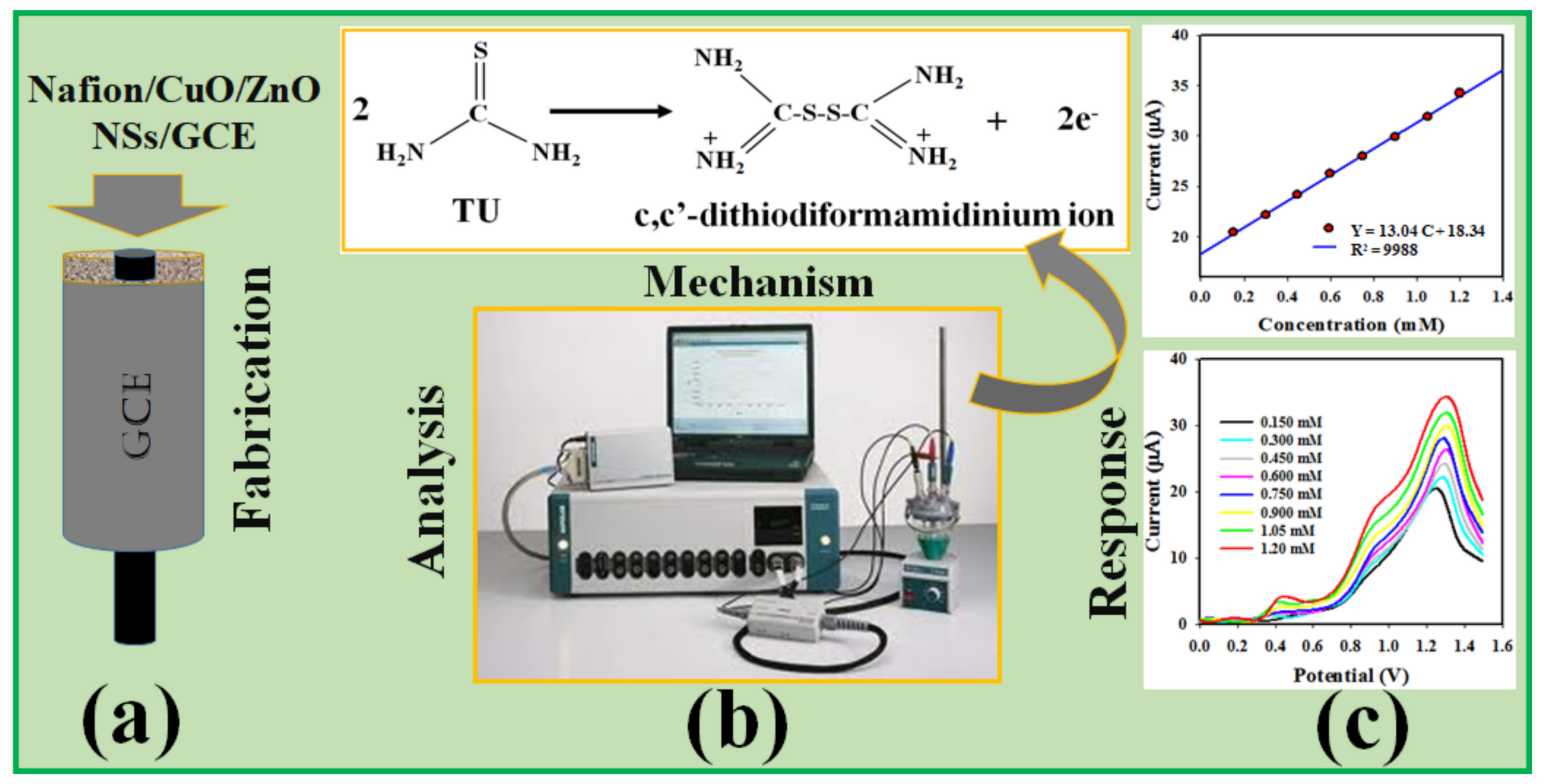
Schematic representation of electrochemical analyses in the detection of TU. (**a**) Modification of GCE with CuO/ZnO NSs. (**b**) Instrumentation of assembled sensor probe to perform the differential pulse voltammeric analysis of TU to c,c’–dithiodiformamidinium. (**c**) The recorded analysis data.

The proposed electro-chemical oxidation of TU produced a c,c’–dithiodiformamidinium ion (TU^2+^) and free electrons using the Nafion/CuO/ZnO NSs/GCE as a working electrode, as can be seen in Scheme 1. As a result, the conductance of the phosphate buffer medium was amplified and obtained a DPV response. The similar oxidation of TU was analyzed by using voltammetry and potentiostatic coulometry [47,48].

To accomplish the evaluation of similar research works with this study, in Table 1 [10,49,50,51], we illustrate the analytical parameters of TU sensors, such as LDR, LOD and sensitivity. Consequently, the sensor performances of this sensor probe, such as linear dynamic range (LDR), sensitivity and limit of detection (LOD), were found to be significant and excellent compared to published reports [52,53,54,55,56,57,58]. Moreover, other sensor analytical parameters, including sensor stability, reproducibility, response time and analysis of real biological samples, were good and reliable. Finally, we introduce a noble sensor probe based on a binary, doped nanostructure material coated with a conducting coating binder by electrochemical approach for the efficient detection of hazardous chemicals in biological samples for safety purposes in the biomedical and healthcare fields on a broad scale.

### 3.6. Analysis of Real Samples

Finally, the fabricated Nafion/CuO/ZnO NSs/GCE sensor probe for TU detection was used to analyze the collected biological samples, such as human, rabbit and mouse serum, by the DPV method. In this study, the known concentration of TU was added in the phosphate buffer solution at pH 7.0. Then, the collected human urine and blood serum were added into the electrochemical cell to formulate the real samples, which is illustrated in Table 2. Finally, the formulated real samples were analyzed by DPV using the modified GCE (Nafion/CuO/ZnO NSs/GCE). Finally, the responses were compared with the added known concentration of TU. The obtained results are presented in Table 2. The results are quite acceptable and satisfactory.

## 4. Conclusions

In this study, sandwich-type CuO/ZnO NSs were prepared by the hydrothermal method in an alkaline phase. The prepared NSs were characterized in detail by using conventional methods, such as powder XRD, XPS, FESEM, EDS, etc. Then, the fabricated sensor probe based on a sandwich-type Nafion/CuO/ZnO NSs/GCE sensor probe was successfully employed in the selective detection of TU in a phosphate buffer solution (pH 7.0). It showed good sensitivity (0.4122 µA µM^−1^ cm^−2^), as well as a large linear detection range (0.15~1.20 mM) with the lowest detection limit (23.03 ± 1.15 µM). This sensor probe was practically validated with human serum and the obtained results are acceptable and satisfactory. Besides this, we significantly accomplished the assessment of the biological samples with high reproducibility, good stability and short response time. Then, this electro-chemical approach could constitute a turning point for the improvement of portable electro-chemical sensor probe development in real time monitoring for biological analysis for early diagnosis in the biomedical and healthcare fields on a broad scale.

## Figures and Tables

**Table 1:** The comparison of analytical performances of TU sensors based on various nanomaterial sensor probe by electrochemical method.

Modified Electrode	Method	* DOL	*^#^* LDR	Sensitivity	Ref.
CuO/CuE	CV	-	1~8 mM	-	[49]
AuE	CV	0.05 mM	0.05~1 mM	-	[50]
Materials	HPLC	2 µM	10–50 µM	-	[10]
AYP/GCE	CV	3.3 µM	10~940 µM	0.181 µAµM^−1^	[51]
Nafion/CuO/ZnO NSs/GCE	DPV	23.03 µM	0.15~1.20 mM	0.4122 µAµM^−1^cm^−2^	This work

* DL (detection limit), ^#^ LDR (linear dynamic range), µM (micromole), mM (millimole).

**Table 2:** Sensor analyses of real biological samples with Nafion/CuO/ZnO NSs/GCE probe by electro-chemical method.

Method	Real Samples	Added TU Conc. (mM)	Measured TU Conc. ^a^ by Nafion/CuO/ZnO NSs/GCE (mM)			Average Recovery ^b^ (%)	RSD ^c^ (%) (n = 3)
			R1	R2	R3		
DPV	Human urine serum	0.4500	0.4421	0.4397	0.4400	97.91	0.30
	Human urine serum	0.6000	0.5744	0.5821	0.5792	96.43	0.67
DPV	Human blood serum	0.1500	0.1621	0.1657	0.1643	109.56	1.11
	Human blood serum	0.7500	0.7450	0.7612	0.7355	99.63	1.74

^a^ Mean of three repeated determinations (signal-to noise-ratio, 3) with Nafion/CuO/ZnO NSs/GCE. ^b^ Concentration of TU determined/Concentration taken (unit: µM). ^c^ Relative standard deviation value indicates precision among 3 repeated measurements (R1, R2 and R3).

## Data Availability

Data will be available upon request.

## References

[1] De Oliveira A.N., De Santana H., Zaia C.T.B.V., Zaia D.A.M. (2004). A study of reaction between quinones and thiourea: Determination of thiourea in orange juice. J. Food Compos. Anal..

[2] Moragues M.E., Santos-Figueroa L.E., Ábalos T., Sancenón F., Martínez-Máñez R. (2012). Synthesis of a new tripodal chemosensor based on 2,4,6-triethyl-1,3,5-trimethylbencene scaffolding bearing thiourea and fluorescein for the chromo-fluorogenic detection of anions. Tetrahedron Lett..

[3] Chalapathi U., Poornaprakash B., Park S.H. (2017). Effect of thiourea concentration on the growth and properties of CuSnS4 thin films prepared by spray pyrolysis. J. Mater. Sci. Mater. Electron..

[4] Rahman M.M., Alam M.M., Asiri A.M. (2020). Detection of thiourea with ternary Ag_2_O/TiO_2_/ZrO_2_ nanoparticles by electrochemical approach. J. Mater. Sci. Mater. Electron..

[5] Gholap S.V., Dod V.N., Bhuyar S.A., Bharad S.G. (2000). Effect of plant growth regulators on seed germination and seedling growth in aonla (*Phyllanthus emblica* L.) under climatic condition of Akola. Crop Res. Hisar.

[6] Sokkar S.M., Soror A.H., Ahmed Y.F., Ezzo O.H., Hamouda M.A. (2000). Pathological and biochemical studies on experimental hypothyroidism in growing lambs. J. Vet. Med. Ser. B.

[7] Bhide S.V., Deshmuh B.T., Talvelkar B.A., Nagvekar A.S. (2001). Effect of Induced Hypothyroidism on Blood Biochemical Constituents in Goats. Indian Vet. J..

[8] Cooper D.S. (1984). Antithyroid drugs. N. Engl. J. Med..

[9] Kies H.L. (1978). The conductometric titration of thiourea by mercury (II) chloride. Anal. Chim. Acta.

[10] Rethmeier J., Neumann G., Stumpf C., Rabenstein A., Vogt C. (2001). Determination of low thiourea concentrations in industrial process water and natural samples using reversed-phase high-performance liquid chromatography. J. Chromatogr. A.

[11] Kargosha K., Khanmohammadi M., Ghadiri M. (2001). Fourier transform infrared spectrometric determination of thiourea in the presence of sulphur dioxide in aqueous solution. Anal. Chim. Acta.

[12] Chen C., Zhao D., Sun J., Yang X. (2016). A dual-mode signaling response of a Au-NP-fluorescein based probe for specific detection of thiourea. Analyst.

[13] Fan X., Fan Z. (2019). Determination of Thiourea by On–Off Fluorescence Using Nitrogen-Doped Graphene Quantum Dots. Anal. Lett..

[14] Wang G.L., Dong Y.M., Zhu X.Y., Zhang W.J., Wang C., Jiao H.J. (2011). Ultrasensitive and selective colorimetric detection of thiourea using silver nanoprobes. Analyst.

[15] Wang W., Dong Z.Z., Yang C., Li G., Tse Y.C., Leung C.H., Ma D.L. (2017). An iridium (III) complex-based chemosensor for the detection of thiourea in living cells. Sens. Actuators B.

[16] Pedre I., Battaglini F., Delgado G.J.L., Sánchez-Loredo M.G., González G.A. (2015). Detection of thiourea from electrorefining baths using silver nanoparticles-based sensors. Sens. Actuators B.

[17] Rahman M.M., Ahmed J., Asiri A.M. (2018). Thiourea sensor development based on hydrothermally prepared CMO nanoparticles for environmental safety. Biosens. Bioelectron..

[18] Alam M.M., Uddin M.T., Asiri A.M., Rahman M.M., Islam M.A. (2020). Development of reproducible thiourea sensor with binary SnO_2_/V_2_O_5_ nanomaterials by electrochemical method. Arab. J. Chem..

[19] Pedre I., De-Leo L.M., Sánchez-Loredo M.G., Battaglini F., González G.A. (2016). Electrochemical sensor for thiourea focused on metallurgical applications of copper. Sens. Actuators B Chem..

[20] Saharan P., Sharma A.K., Kumar V., Kaushal I. (2019). Multifunctional CNT supported metal doped MnO_2_ composite for adsorptive removal of anionic dye and thiourea sensing. Mater. Chem. Phys..

[21] Li G., Miao P. (2013). Electrochemical Analysis of Proteins and Cells.

[22] Wei Z., Yang Y., Wang J., Zhang W., Ren Q. (2018). The measurement principles, working parameters and configurations of voltammetric electronic tongues and its applications for foodstuff analysis. J. Food Eng..

[23] Zhang J., Zeng D., Zhu Q., Wu J., Huang Q., Zhang W., Xie C. (2016). Enhanced room temperature NO_2_ response of NiO-SnO_2_ nanocomposites induced by interface bonds at the p-n heterojunction. Phys. Chem. Chem. Phys..

[24] Vuong N.M., Chinh N.D., Huy B.T., Lee Y. (2016). CuO-Decorated ZnO Hierarchical Nanostructures as Efficient and Established Sensing Materials for H_2_S Gas Sensors. Sci. Rep..

[25] Hussain M.M., Asiri A.M., Rahman M.M. (2020). Non-enzymatic simultaneous detection of acetylcholine and ascorbic acid using ZnO·CuO nanoleaves: Real sample analysis. Microchem. J..

[26] Alam M.M., Asiri A.M., Uddin M.T., Inamuddin, Islam M.A., Awual M.R., Rahman M.M. (2019). One-step wet-chemical synthesis of ternary ZnO/CuO/Co_3_O_4_ nanoparticles for sensitive and selective melamine sensor development. New J. Chem..

[27] Khan A.A.P., Khan A., Rahman M.M., Asiri A.M., Oves M. (2017). Sensor development of 1,2 Dichlorobenzene based on polypyrole/Cu-doped ZnO (PPY/CZO) nanocomposite embedded silver electrode and their antimicrobial studies. Int. J. Biol. Macromol..

[28] Ahmad R., Tripathy N., Ahn M.S., Bhat K.S., Mahmoudi T., Wang Y., Yoo J.Y., Kwon D.W., Yang H.Y., Hahn Y.B. (2017). Highly Efficient Non-Enzymatic Glucose Sensor Based on CuO Modified Vertically-Grown ZnO Nanorods on Electrode. Sci. Rep..

[29] Yoon S.S., Ramadoss A., Saravanakumar B., Kim S.J. (2014). Novel Cu/CuO/ZnO hybrid hierarchical nanostructures for non-enzymatic glucose sensor application. J. Electroanal. Chem..

[30] Rybarczyk P., Kawalec-Pietrenko B. (2021). Simultaneous Removal of Al, Cu and Zn Ions from Aqueous Solutions Using Ion and Precipitate Flotation Methods. Processes.

[31] Quirino M.R., Lucena G.L., Medeiros J.A., dos Santos I.M.G., de Oliveira M.J.C. (2018). CuO Rapid Synthesis with Different Morphologies by the Microwave Hydrothermal Method. Mater. Res..

[32] Zhu D., Wang L., Yu W., Xie H. (2018). Intriguingly high thermal conductivity increment for CuO nanowires contained nanofuids with low viscosity. Sci. Rep..

[33] Muhammad W., Ullah N., Haroon M., Abbasi B.H. (2019). Optical, morphological and biological analysis of zinc oxide nanoparticles (ZnO NPs) using *Papaver somniferum* L.. RSC Adv..

[34] Zak A.K., Razali R., Majid W.H.A., Darroudi M. (2011). Synthesis and characterization of a narrow size distribution of zinc oxide nanoparticles. Int. J. Nanomed..

[35] Alam M.M., Rahman M.M., Uddin M.T., Asiri A.M., Inamuddin, Chani M.T.S., Islam M.A. (2020). Development of L-glutamic acid biosensor with ternary ZnO/NiO/Al_2_O_3_ nanoparticles. J. Lumin..

[36] Alam M.M., Uddin M.T., Asiri A.M., Awual M.R., Fazal M.A., Rahman M.M., Islam M.A. (2020). Fabrication of selective L-glutamic acid sensor in electrochemical technique from wet-chemically prepared RuO_2_ doped ZnO nanoparticles. Mater. Chem. Phys..

[37] Alam M.M., Mukhlish M.Z.B., Tazrin A., Jui N.A., Asiri A.M., Rahman M.M., Islam M.A., Uddin M.T. (2020). A novel highly selective electrochemical chlorobenzene sensor based on ternary oxide RuO_2_/ZnO/TiO_2_ nanocomposites. RSC Adv..

[38] Alam M.M., Asiri A.M., Uddin M.T., Rahman M.M., Islam M.A. (2020). An alternative electrochemical approach for toluene detection with ZnO/MgO/Cr_2_O_3_ nanofibers on a glassy carbon electrode for environmental monitoring. RSC Adv..

[39] Alam M.M., Asiri A.M., Rahman M.M., Islam M.A. (2020). Fabrication of sensitive D-fructose sensor based on facile ternary mixed ZnO/CdO/SnO_2_ nanocomposites by electrochemical approach. Surf. Interfaces.

[40] Alam M.M., Rahman M.M., Asiri A.M., Fazal M.A. (2021). A reliable electrochemical approach for detection of testosterone with CuO-doped CeO_2_ nanocomposites coated glassy carbon electrode. J. Mater. Sci. Mater. Electron..

[41] Rahman M.M., Alam M.M., Asiri A.M., Opo F.A.D.M. (2020). Fabrication of selective and sensitive chemical sensor probe based on ternary nano-formulated CuO/MnO_2_/Gd_2_O_3_ spikes by hydrothermal approach. Sci. Rep..

[42] El-Nahrawy A.M., Elzwawy A., Alam M.M., Hemdan B.A., Asiri A.M., Karim M.R., Hammad A.B.A., Rahman M.M. (2021). Synthesis, structural analysis, electrochemical and antimicrobial activities of copper magnesium zirconosilicate (Cu_20_Mg_10_Si_40_Zr_(30-x)_O_:(x = 0,5,7,10)_ Ni^2+^) nanocrystals. Microchem. J..

[43] Zeid E.F.A., Nassar A.M., Husseind M.A., Alam M.M., Asiri A.M., Hegazy H.H., Rahman M.M. (2020). Mixed oxides CuO-NiO fabricated for selective detection of 2-Aminophenol by electrochemical approach. J. Mater. Res. Technol..

[44] Asiri A.M., Adeosun W.A., Marwani H.M., Rahman M.M. (2020). Homopolymerization of 3-aminobenzoic acid for enzyme-free electro catalytic assay of nitrite ions. New J. Chem..

[45] Alizadeh T., Azizi S. (2016). Graphene/graphite paste electrode incorporated with molecularly imprinted polymer nanoparticles as a novel sensor for differential pulse voltammetry determination of fluoxetine. Biosens. Bioelectron..

[46] Ahmed J., Rakib R.H., Rahman M.M., Asiri A.M., Siddiquey I.A., Islam S.S.M., Hasnat M.A. (2019). Electrocatalytic Oxidation of 4-Aminophenol Molecules at the Surface of an FeS_2_/Carbon Nanotube Modified Glassy Carbon Electrode in Aqueous Medium. ChemPlusChem.

[47] Kirchnerova J., Purdy W.C. (1981). The mechanism of the electrochemical oxidation of thiourea. Anal. Chim. Acta.

[48] Yan M., Liu K., Jiang Z. (1996). Electrochemical oxidation of thiourea studied by use of in situ FTIR spectroscopy. J. Electroanal. Chem..

[49] Manea F., Radovan C., Schoonman J. (2006). Amperometric determination of thiourea in alkaline media on a copper oxide–copper electrode. J. Appl. Electrochem..

[50] Lee J.W., Mho’ S., Pyun C.H., Yeo I.H. (1994). Flow Injective Determination of Thiourea by Amperometry. Bull. Korean Chem. Soc..

[51] Jodan I., Wantala K., Amini N., Shahmoradi B., Ghaslani M., Lee S.M., Yang J., Puttaiah S.H. (2020). Fabrication of a sensitive electrochemical sensor based on Ag nanoparticles and alizarin yellow polymer: Application to the detection of an environmental pollutant thiourea. Korean J. Chem. Eng..

[52] Shabik M.F., Begum H., Rahman M.M., Marwani H.M., Hasnat M.A. (2020). Heterogeneous kinetics of thiourea electro-catalytic oxidation reactions on palladium surface in the aqueous medium. Chem. Asian J..

[53] Subhan M.A., Jhuma S.S., Saha P.C., Ahmed J., Asiri A.M., Rifat T.P., Raihan T., Azad A.K., Rahman M.M. (2020). Photocatalysis, Enhanced Anti-bacterial Performance and Discerning Thiourea Sensing of Ag_2_O·SnO_2_·TiO_2_. J. Environ. Chem. Eng..

[54] Rahman M.M., Hussain M.M., Asiri A.M. (2016). A novel approach towards the hydrazine sensor development by SrO·CNT nanocomposites. RSC Adv..

[55] Rahman M.M., Abu-Zied B.M., Hasan M.M., Asiri A.M., Hasnat M.A. (2016). Fabrication of selective 4-aminophenol sensor based on H-ZSM-5 zeolites deposited silver electrodes. RSC Adv..

[56] Mamun M.R.A., Karim M.R., Rahman M.M., Asiri A.M., Torii S. (2016). Methane enrichment of biogas by carbon dioxide fixation with calcium hydroxide and activated carbon. J. Taiwan Inst. Chem. Eng..

[57] Arshad M.N., Sheikh T.A., Rahman M.M., Asiri A.M., Marwany H.M., Awual M.R. (2017). Fabrication of cadmium ionic sensor based on (E)-4-Methyl-N′-(1-(pyridin-2-yl)ethylidene) benzenesulfonohydrazide (MPEBSH) by electrochemical approach. J. Organomet. Chem..

[58] Subhan M.A., Saha P.C., Rahman M.M., Akand M.A.R., Asiri A.M., Al-Mamun M. (2017). Enhanced photocatalytic and chemical sensor development based on ternary B_2_O_3_·Zn6Al_2_O_9_·ZnO nanomaterials for environmental safety. New J. Chem..

